# Non-Contact Stable Arterial Pulse Measurement Using mmWave Array Radar

**DOI:** 10.3390/bioengineering11121203

**Published:** 2024-11-28

**Authors:** Fanglin Geng, Zhongrui Bai, Hao Zhang, Changyu Liu, Peng Wang, Zhenfeng Li, Lidong Du, Xianxiang Chen, Zhen Fang

**Affiliations:** 1 Aerospace Information Research Institute, Chinese Academy of Sciences (AIRCAS), Beijing 100094, China; gengfanglin20@mails.ucas.ac.cn (F.G.); zhongrui.bai@sjtu.edu.cn (Z.B.); zhanghao190@mails.ucas.ac.cn (H.Z.); liuchangyu21@mails.ucas.ac.cn (C.L.); wangpeng01@aircas.ac.cn (P.W.); lizhenfeng@aircas.ac.cn (Z.L.); lddu@mail.ie.ac.cn (L.D.); chenxx@aircas.ac.cn (X.C.); 2 School of Electronic, Electrical and Communication Engineering, University of Chinese Academy of Sciences, Beijing 101408, China; 3 School of Electronic Information and Electrical Engineering, Shanghai Jiao Tong University, Shanghai 200240, China; 4 Personalized Management of Chronic Respiratory Disease, Chinese Academy of Medical Sciences, Beijing 100700, China

**Keywords:** non-contact pulse wave measurement, multiple site arteries, large-array millimeter-wave radar, target localization and focusing

## Abstract

Pulse signals can serve as important indicators of one’s cardiovascular condition. However, capturing signals with stable morphology using radar under varying measurement periods remains a significant challenge. This paper reports a non-contact arterial pulse measurement method based on mmWave radar, with stable signals achieved through a range–angle focusing algorithm. A total of six subjects participated in the experiment, and the results showed that, under different measurement times, the pulse morphology of the same body part for each subject had good consistency, reaching a correlation of over 0.84, and four selected pulse signs remained stable. This is a quantitative assessment revealing a high correlation in pulse morphology measured by radar over different periods. In addition, the influence of array size and measurement distance was analyzed, providing a reference of array selection for research work with different requirements. This work offers an effective reference framework for long-term pulse measurement using radar technology.

## 1. Introduction

Continuous tracking of cardiovascular status is important for assessing health status [1,2]. Therefore, it is very important to construct a system for long-term monitoring of vascular status [2]. The pulse wave carries valuable information about cardiovascular health. As blood flows through vessels, changes in arterial compliance lead to variations in pulse waveforms at different measurement sites [3]. Therefore, the pulse wave can be used as a key indicator for assessing cardiovascular health status [4,5,6].

The earliest pulse wave monitoring method was invasive measurement, in which a catheter was placed in the artery to observe continuous pulse wave signals. Since the beginning of the last century, in order to overcome the many inconveniences of invasive sensors [7], many ingenious methods and devices for the non-invasive acquisition of pulse signals have been proposed. Among them, one of the most commonly used methods to date is the tension method. However, although the pressure sensor can provide accurate pressure pulse wave measurements, its sensitivity to motion and dependence on operator skills make it less suitable for clinical screening applications. In addition, some other new contact monitoring methods have emerged in recent years, including photoplethysmography (PPG) sensors [8,9,10,11], microelectromechanical system sensors [12,13,14,15], and combinations of cardiopulmonary function graphs, electrocardiographs (ECGs), and impedance cardiogram (ICG) sensors [16,17,18,19,20]. However, these methods rely on direct contact with the patient’s skin, which may cause discomfort and pressure, especially for vulnerable populations such as infants, the elderly, and patients with dementia. In addition, surface loading effects and body fluids (such as sweat) can reduce the accuracy of contact measurements [21]. Moreover, contact sensors may require fixed measures to ensure monitoring, which reduces the patient’s quality of life, further limiting the patient’s compliance with contact sensors and affecting the patient’s enthusiasm for use [22,23].

In recent years, radar-based non-contact vital sign detection has attracted widespread attention in the microwave and medical communities [24,25,26,27,28]. Based on the Doppler phase modulation effect, radar can sense physiological motion without installing any probes on the human body, which makes vital sign detection more flexible and attractive for long-term monitoring [29]. Using the displacement-phase modulation effect of electromagnetic waves, the vibration of the skin surface caused by the propagation of pulse waves in blood vessels can be measured. Therefore, measurement methods based on mmWave radar technology can provide new ideas about non-contact pulse wave monitoring and cardiovascular status analysis, which has good practical prospects in terms of comfort. The user’s pulse wave can be captured by radar in a non-contact manner without wearing any sensors. This monitoring method brings the significant benefit of zero interference. Users can complete pulse wave monitoring in a non-sensory way while sleeping or working.

Radar-based technology has been increasingly used to monitor vital signs such as heartbeat and breathing [30,31,32,33]. In addition, there are also some methods for monitoring pulse wave signals. Buxi et al. attached the radar antenna directly to the skin of the subject to monitor the chest pulse wave signal [11]; however, the contact measurement method is inconvenient for the user and is not suitable for daily monitoring. Johnson et al. embedded the radar antenna in a wrist-worn device to monitor the radial artery pulse wave [34]; the radial artery is a peripheral artery. Compared with the carotid artery, thoracic aorta, and abdominal aorta, which are close to the center, the peripheral part contains more smooth muscle and has a relatively weaker correlation with the cardiovascular system. So, the proximal aorta is a more suitable monitoring site [8,35]. Oyamada et al. used array radar to measure the arterial pulse wave at the back and calf of the human body and used the laser displacement sensor as a reference to verify the accuracy of the displacement signal measured by the radar [36]. This part of the work involved relevant experiments on two subjects to verify the accuracy of the radar monitoring of the skin surface displacement signal, but did not study the periodicity and regularity of the pulse wave signal morphology. Will et al. conducted relevant research on the morphology of pulse wave signals. They studied the effects of filtering and antenna characteristics on signals by monitoring the neck and chest separately, using these findings to explain differences in signal morphology observed by different research teams [37]. This article points out that different teams measured the radar signals of different morphologies, which are related to different filtering methods and antenna characteristics. Narrowband filtering can cause signal distortion. When the antenna is far away from the target, it is difficult to focus on a specific area. For example, in the chest position, various cardiac physiological effects will be superimposed. The radar captures the superimposed movement of the atria, ventricles, and chest arteries, which will result in different signal waveforms. In addition, Pour et al. pointed out that the morphology of the pulse wave signal measured by the radar is affected by the position and direction of the heart in the chest cavity, so it is difficult to monitor highly repeatable signals [38].

The aforementioned studies primarily focus on the differences in pulse wave signal morphology, offering explanations for the inconsistencies in waveform morphology observed at different time periods. To the best of the authors’ knowledge, no prior research has quantitatively evaluated the repeatability and consistency of pulse wave signal morphology measured by radar across different body parts. We attribute the possible reasons to two key aspects: hardware and algorithm. On the hardware side, limitations stem from the angular resolution of small-array radars. On the algorithmic side, in most experimental radar studies, the energy of the radar beam is not sufficiently focused.

Therefore, starting from these two aspects, we used MIMO array radar and combined it with range–angle target focusing processing to obtain the arterial pulse wave signals of the subject’s neck, chest, and abdomen, and analyzed the stability and repeatability of the signals in each part during different measurement periods. In addition, we further designed experiments to discuss the influence of array size and measurement distance on pulse wave acquisition, which provides an effective reference scheme for non-contact long-term measurement of arterial pulse waves based on array radar. In the following sections, we briefly explain the fundamental principles and methods of this work, detailing the signal processing procedures and related experimental setups. Based on this foundation, we present experimental results to validate the performance. Finally, we discuss the research findings and outline the next steps for future work.

## 2. Methods

### 2.1. Principle and Method of Arterial Pulse Monitoring Based on Radar

Assuming that the distance between the radar and the subject is *R*, the beam is vertically directed toward the human body, and the subject has no random body shaking except for breathing and heartbeat movements. As the pulse wave travels through the blood vessels, it induces displacement of the skin surface along its propagation path. The principle of mmWave radar detecting skin surface vibration is as follows.

We assume that the radar-transmitted signal is

(1) $$X_{T}=A_{T}\cos\left(2\pi f_{1}t+\pi \frac{B}{T_{c}}t^{2}+\theta \left(t\right)\right).$$

Among them, $f_{1}$ is the starting frequency of the transmitted signal, $T_{c}$ is the duration of a single transmitted signal, $A_{T}$ is the amplitude of the transmitted signal, $\frac{B}{T_{c}}$ is the slope of the signal, and θ(t) is the noise.

Assuming the initial distance of the radar from the human body is *R* and the skin surface vibration is C(t), then the total distance is R(t)=R+C(t). Therefore, the time delay is

(2) $$t_{d}=\frac{2R(t)}{c}.$$

where *c* is the speed of light. Then, the received signal can be obtained as

(3) $$A_{R}\cos\left(2\pi f_{1}\left(t-t_{d}\right)+\pi \frac{B}{T_{c}}{\left(t-t_{d}\right)}^{2}+\theta \left(t-t_{d}\right)\right).$$

After I/Q channel demodulation, the frequency and phase of the intermediate frequency signal is, respectively,

(4) $$f=\frac{2BR}{cTc}.$$

(5) $$\phi \left(t\right)=\frac{4\pi R(t)}{\lambda }.$$

Therefore, according to Equation (5), the periodic changes in skin surface vibration can be obtained by observing the phase changes in the radar echo signal.

### 2.2. Effect of Radar Array Size on Pulse Wave Measurement

The size of the radar antenna array influences the extent of the target monitoring area. Upon reviewing previous studies, we found that most employed small-array radars, while larger arrays, particularly 8-element arrays, were the most commonly used in other cases. For instance, in the studies referenced in [39,40], single-transmitter, single-receiver radar, and 2-transmitter, 2-receiver radar were used for heart rate measurement and blood pressure monitoring, respectively. Meanwhile, in [36], an 8-element radar array was employed for pulse wave monitoring (although some studies used 12-element arrays, the effective target dimension was equivalent to 8 arrays). Therefore, we take an 8-array radar as an example to analyze the differences between different arrays of radar in terms of angular resolution and monitoring area. Taking the uniform linear array (ULA) in the target dimension as an example, the angular resolution of the linear array radar depends on the size of the antenna array and the wavelength of the signal. For the ULA antenna, the angular resolution can be calculated by Equation (6):

(6) $$\theta_{\min}=\frac{\lambda }{N\cdot d\cdot \cos(\theta )}.$$

where $\theta_{\min}$ is the angular resolution, λ is the wavelength of the signal, *N* is the number of antenna elements, *d* is the spacing between antenna elements, and θ is the angle of the target direction relative to the antenna array.

For an 8-array linear array radar, if the spacing between antenna elements is half a wavelength ($d=\frac{\lambda }{2}$), the formula is simplified to

(7) $$\theta_{\min}=\frac{2}{N\cdot \cos(\theta )}.$$

In this case, assuming the target is directly in front (*θ* = 0), then the angular resolution is $\theta_{\min}=\frac{2}{8}=14.3^{\circ }$. Therefore, for an 8-array linear array radar, under ideal conditions, its angular resolution is approximately 14.3 degrees.

Considering a radar with a larger array, such as the 16 × 12 MIMO array radar used in this paper, the number of virtual arrays in the horizontal direction reaches 86, and the spacing between antenna array elements is half a wavelength. Ideally, assuming that the target is located directly in front, the angular resolution of the radar with an array of 86 is $\theta_{\min}=\frac{2}{86}=1.3^{\circ }$. Compared with the 8-array radar, the beam width is narrowed approximately 10 times.

**Figure 1:**
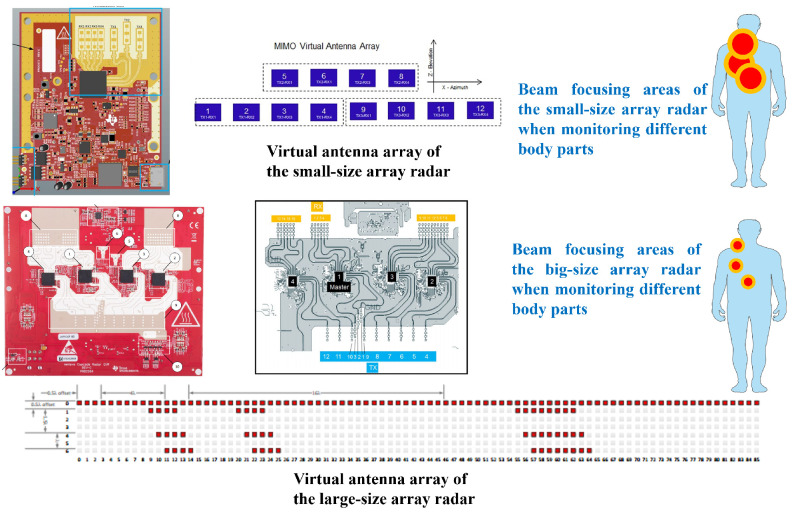
Schematic diagram comparing the monitoring areas of a large-array radar and a small-array radar, the numbers in the small array represent the 12 transceiver paths, while the numbers in the large array indicate the indices of the transmit and receive antennas (in fact, the shape of the light spot is not a circle, but a strip-shaped spot. Here, the light spot is drawn as a circle for the convenience of display and explanation).

Next, consider the difference in monitoring area between a large-array radar and a small-array radar. The size of the monitoring area corresponds to the area of the spot formed by the radar transmission beam in the target region, which can be calculated by the angular resolution of the antenna array. Assume that the target is 50 cm away from the radar and facing the direction of the radar. For the 8-array radar, the beam spot width on the target surface is about 126 mm; for the 86-array radar, the beam spot width on the target surface is about 12 mm. As shown in Figure 1, the monitoring area of the small-array radar is larger and cannot accurately focus on the target measurement area, so it is more likely to contain mixed signals from multiple parts. In comparison, the large-array radar can focus on and locate the target part more accurately, thereby obtaining a more accurate and stable signal.

### 2.3. Signal Processing Flow

**Figure 2:**
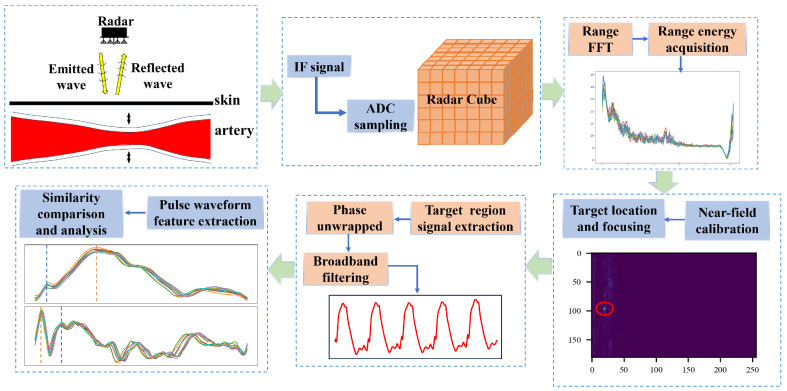
Radar signal processing chain. The arrows indicate the sequence of the signal processing flow, the lines in different colors represent pulse wave signals from different cycles, and the target with the highest energy in the heatmap is marked with a red circle.

The main signal processing flow is shown in Figure 2.

**Figure 3:**
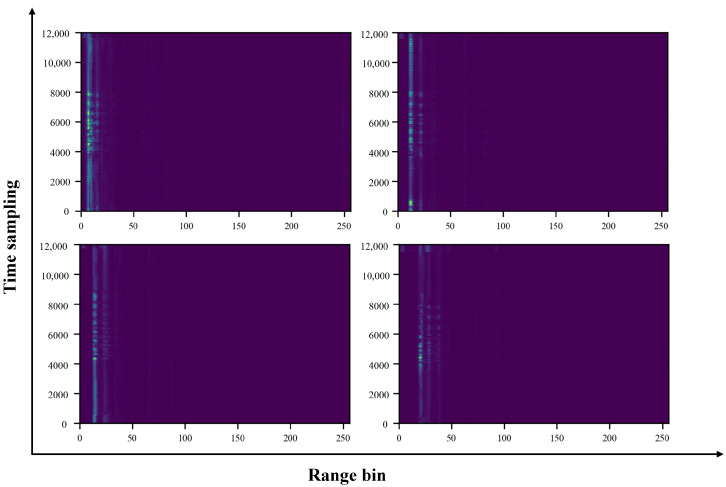
Time–range heat maps at measurement distances of 20 cm, 40 cm, 50 cm, and 70 cm using mmWave radar. The greater the energy, the brighter it appears.

(1) One-dimensional FFT and target range bin selection: Firstly, a distance dimension FFT is performed on each complex received signal from a total of 16 × 12 channels to obtain the distance distribution of the measured scene, so as to select the range bin where the target is located. Figure 3 shows the distance distribution of the target scene taking thoracic artery location as an example, and shows the time–range heat map of the mmWave radar at different measurement distances.

(2) Phase extraction based on ArcTanGent demodulation: Phase unwrapping is performed using the ArcTanGent demodulation method. The principle of ArcTanGent demodulation is to use the orthogonality of two orthogonal baseband signals and divide the orthogonal branch signal by the in-phase branch signal. The phase information is extracted by solving the ArcTanGent as Equation (8).

(8) $$\Phi \left(t\right)=\arctan\left[\frac{B_{Q}\left(t\right)}{B_{I}\left(t\right)}\right]=\frac{4\pi x(t)}{\lambda }+\phi .$$

The main problem of the ArcTanGent method is phase discontinuity. The phase compensation method is used to solve this problem. The phase difference between the two sets of data before and after is compared. When the phase difference is greater than 180° or less than −180°, an integer multiple of 180 is subtracted or added.

(3) Near-field calibration and acquisition of the range–angle heat map: In previous studies, most teams used small-array radars to monitor physiological parameters, assuming that the target is in the far field, so the electromagnetic waves between the target and multiple antennas can be considered parallel. However, with large arrays, the distance between the target and the antenna no longer satisfies the far-field assumption, and the parallel wave theory is no longer applicable. When using traditional FFT technology for angle estimation, an obvious phase error will be generated. Then, near-field correction is required to eliminate the phase difference between the propagation paths of each antenna.

**Figure 4:**
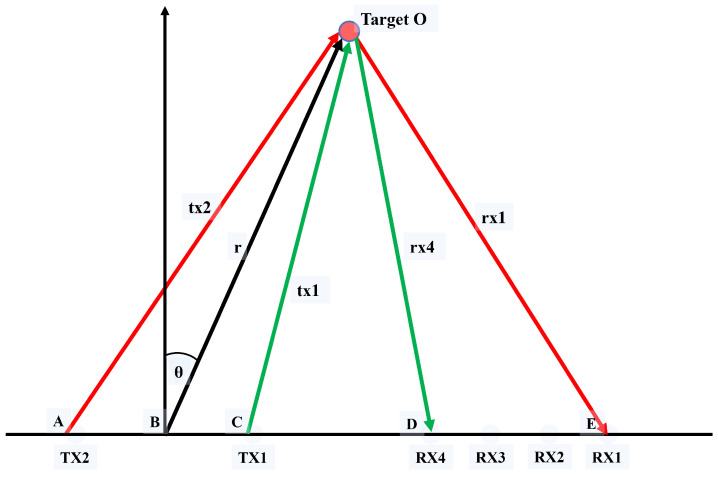
Phase difference between the transmission and reception paths in the near field.

As shown in Figure 4, assuming there is a 2-transmit and 4-receive antenna array, the distance between the target and the antenna array does not meet the far-field assumption. The phase difference between the path TX1-target O-RX4 and the path TX2-target O-RX1 is the most obvious. This phase difference will cause a false peak to appear on the angle FFT, thus forming a false target. To reduce the impact of this error, it is necessary to correct the phase error between the different transmitting and receiving antenna paths.

Assuming that the target is located at an angle θ, the propagation path lengths of the antennas, tx1, tx2, rx4, and rx1, can be expressed as

(9) $$tx1=\sqrt{r^{2}+{\bar{\mathrm{BC}}}^{2}-2r\bar{\mathrm{BC}}\cos\left(\pi /2-\theta \right)}.$$

(10) $$tx2=\sqrt{r^{2}+{\bar{\mathrm{BA}}}^{2}-2r\bar{\mathrm{BA}}\cos\left(\pi /2+\theta \right)}.$$

(11) $$rx4=\sqrt{r^{2}+{\bar{\mathrm{BD}}}^{2}-2r\bar{\mathrm{BD}}\cos\left(\pi /2-\theta \right)}.$$

(12) $$rx1=\sqrt{r^{2}+{\bar{\mathrm{BE}}}^{2}-2r\bar{\mathrm{BE}}\cos\left(\pi /2-\theta \right)}.$$

It follows that the phase difference between the two paths is

(13) $$\phi \left(k,r\right)=\frac{2\pi }{\lambda }\left\{\left(tx2+rx1\right)-\left(tx1+rx4\right)\right\}.$$

With 1 degree as the angle step unit and 1 range as the distance step unit, the phase error at different angles and distances in space is calculated, and it is used as the correction of the radar raw data to obtain an accurate range–angle heat map. The specific calculation steps are as follows: (a) According to the layout of the MIMO array, a coordinate system is established to determine the coordinate positions of transmitting antennas and receiving antennas. As shown in Figure 4, the position of TX0 is used as the coordinate origin, and the coordinate positions of the remaining antennas are determined according to the distance arrangement between the antennas. (b) According to the coordinate position of each transmitting antenna and receiving antenna, with 1 degree as the step, the angle range is 0–180 degrees, r is from the 1st range to the 256th range, and the path TX0-RX0 is used as the reference. According to Formula (5), the phase difference between the remaining transceiver paths and the reference path is calculated to obtain a phase difference matrix of 86 (number of arrays) × 181 (number of angle bins) × 256 (number of range bins). (c) The phase difference matrix is compensated to the radar receiving signal to obtain the corrected signal and the range–angle heat map, and then the target is positioned and focused.

The advantage of near-field calibration is that the signals of all distances and angles are positioned and focused. If the signal is phase unwrapped solely based on the range where the target is located, the resulting signal is actually an omnidirectional signal within that range bin. When the measurement distance is shorter, the influence of non-target directions is minimal, or the signal’s morphological details can be overlooked for frequency measurements such as heart rate or pulse rate. However, for precise waveform morphology analysis, it is essential to further locate and focus on the target region to eliminate signal interference from non-target directions.

(4) Signal similarity analysis: In order to compare the stability, repeatability, and periodicity of the pulse wave signals monitored under different conditions for the same subject, we calculated the correlation between the overall signals and the stability of the waveform characteristics. Starting from the waveform characteristics of the original signal and the first-order signal, the stability analysis of the pulse wave signal under different measurement conditions was carried out. The Pearson correlation coefficient was used to measure the correlation between two variables and was selected as an index of the similarity of the overall waveform shape; the calculation method is as in Equation (14):

(14) $$r=\frac{\sum_{i=1}^{n}\left(X_{i}-\bar{X}\right)\left(Y_{i}-\bar{Y}\right)}{\sqrt{\sum_{i=1}^{n}{\left(X_{i}-\bar{X}\right)}^{2}}\sqrt{\sum_{i=1}^{n}{\left(Y_{i}-\bar{Y}\right)}^{2}}}.$$

**Figure 5:**
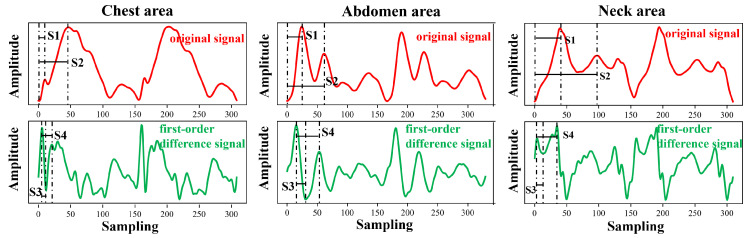
Arterial pulse wave characteristics of the chest, abdomen, and neck area for Subject 1, where, S1–S4 represent four pulse wave features.

**Figure 6:**
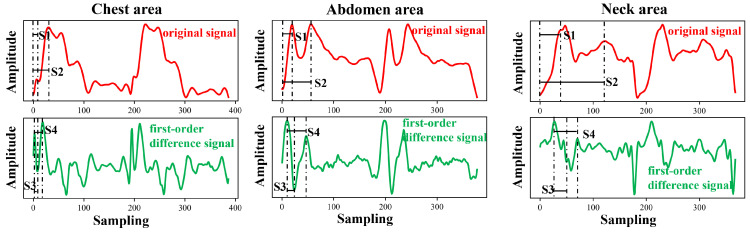
Arterial pulse wave characteristics of the chest, abdomen, and neck area for Subject 2, where, S1–S4 represent four pulse wave features.

Each cycle of the pulse wave signal was interpolated to the same length using the uniform interpolation method, and the correlation analysis of the signal of each cycle was performed according to the Pearson correlation coefficient method. Four waveform features of the original signal and the first-order differential signal were selected to evaluate the stability of the four waveform features under different measurement conditions. Figure 5 and Figure 6 show the pulse wave waveform features selected from the chest, abdomen, and neck regions of two subjects, respectively. The four features of each part are represented by S1, S2, S3, and S4.

## 3. Experimental Setup and Results

Hardware: Texas Instruments’ cascaded mmWave radar device MMWCAS-RF-EVM was used to monitor pulse waves in various parts. The radar operates at a frequency of 76–81 GHz and is a frequency-modulated continuous-wave (FMCW) radar. The radar was connected to a laptop for data collection.

Radar settings: The cascade board includes four AWR2243 devices, each with three transmit antennas and four receive antennas, so the entire device has a total of 12 transmit antennas and 16 receive antennas, collecting data from 192 channels. The antenna transmission parameter settings are shown in Table 1. The ADC sampling rate was set to 7000 ksps. The chirp duration was set to 35 µs. During each chirp duration, the FMCW radar collected 256 samples. The antenna transmission was set to TDM mode. The overall sampling rate of the pulse wave signals was 200 Hz, which is sufficient to achieve pulse wave recovery.

**Figure 7:**
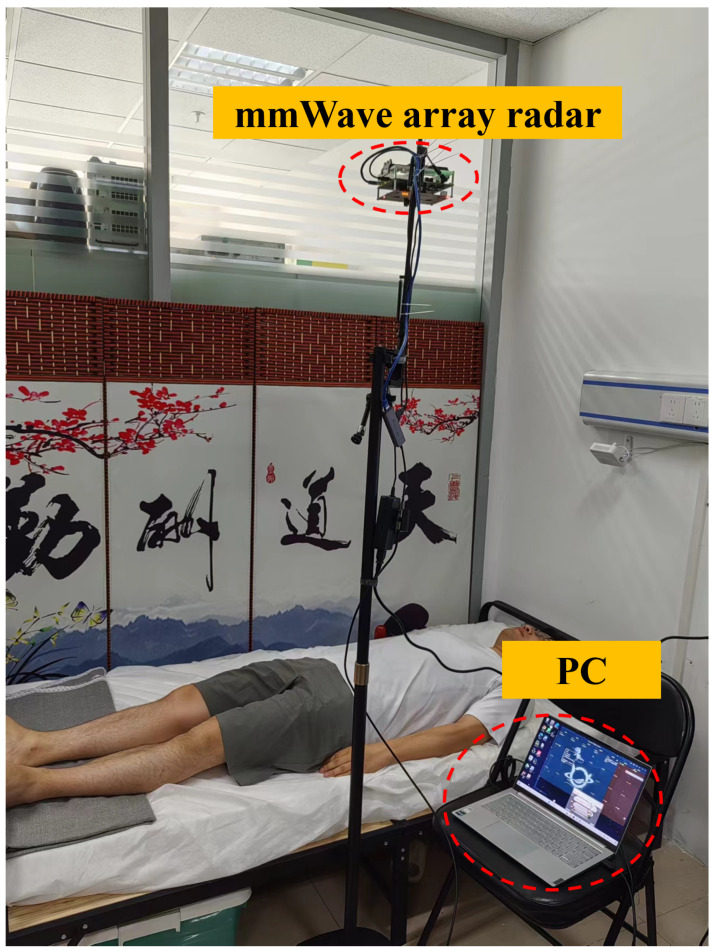
Experimental setup display.

Experimental setup: As shown in Figure 7, each subject was asked to lie flat on the bed. When monitoring the local pulse wave, the radar was aimed at the target monitoring part, 30 cm away from the subject (the distance here is somewhat closer, in order to obtain a more accurate pulse wave signal for reference in subsequent research). During the measurement process, the subject maintained a micro-breath holding state to obtain a more accurate pulse wave morphology reference.

A total of six subjects with normal heart conditions volunteered to participate in the experiment. The experiment was conducted indoors at room temperature and with normal ambient light conditions. Each subject participated in two experimental sessions, conducted one month apart. On each day of the experiment, 12 data sets were collected: 3 sets targeting the neck artery, 3 sets targeting the chest artery, and 3 sets targeting the abdominal artery. Each set included two collections from the same area. Each data collection period lasted 10 s and contained approximately 10 pulse cycles. The signal processing algorithm was implemented on a laptop computer using Python. After the intermediate frequency signal was sampled by the ADC, the FFT of the distance dimension was first performed, and the target range area was selected by calculating the energy of all ranges; the target part was positioned and focused by the range–angle focusing algorithm, the signal of the target part was extracted, and the target pulse wave signal was obtained by phase unwrapping and Butterworth filtering. Here, we did not select a Narrowband filter, but a 0.3–30 Hz broadband filter to retain more pulse wave signal features.

**Figure 8:**
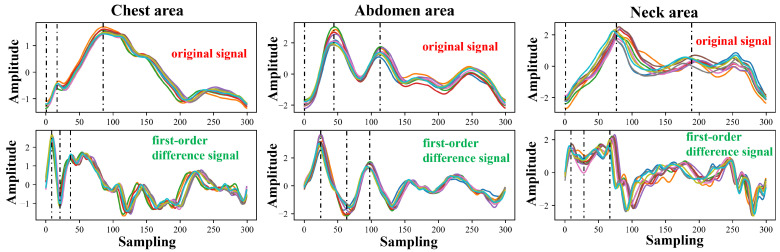
The morphology and characteristics of pulse wave signals at various parts of Subject 1 in the same experiment (randomly selected 10 cycles measured on the same day, identified using different colors).

**Figure 9:**
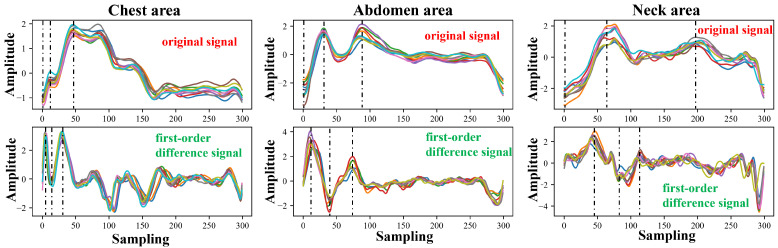
The morphology and characteristics of pulse wave signals at various parts of Subject 2 in the same experiment (randomly selected 10 cycles measured on the same day, identified using different colors).

In Figure 8 and Figure 9, we plot the pulse wave signals of different parts of Subject 1 and Subject 2 and the first-order differential signals of each part in the same experiment (the experiments were conducted on the same day). During the same test, the subject could move their head or body slightly, and the position of the mmWave radar remained unchanged. It can be seen from the figure that in the experiment on the same day, the pulse wave signals of various parts have a high similarity. Among these, the pulse wave signals from the chest and abdomen are relatively smooth, while those from the neck exhibit more fluctuations, which are particularly evident in the first-order differential signal. However, the overall signal morphology trend still has a high correlation. In addition, we also marked the four features of the signal cluster, S1, S2, S3, and S4. It can be seen that the signal features remain stable in the same experiment.

**Figure 10:**
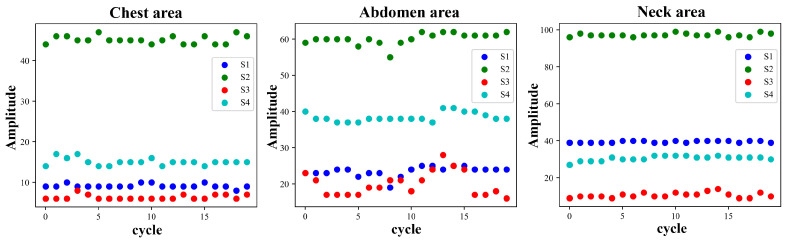
Changes in pulse wave signal characteristics of the chest, abdomen, and neck region of Subject 1 (randomly selected 10 cycles measured on the same day).

**Figure 11:**
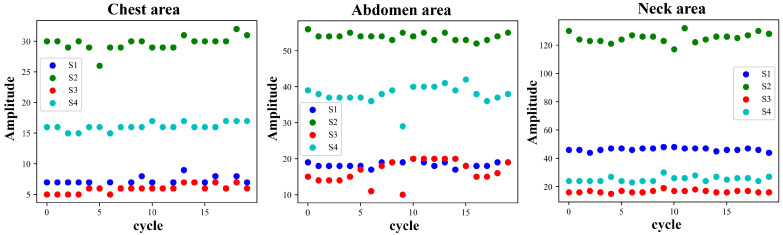
Changes in pulse wave signal characteristics of the chest, abdomen, and neck region of Subject 2 (randomly selected 10 cycles measured on the same day).

Figure 10 and Figure 11 show the changing trends of the four pulse wave characteristics of the neck, chest, and abdomen of Subject 1 and Subject 2 in the experiment on the same day. It can be observed that the pulse wave characteristics of each area remained stable.

The overall morphological similarity of the signals of various body parts for every subject measured in the experiment on the same day is compared, and the results are shown in Table 2. Across all subjects during the same day of the experiment, the average similarity of pulse wave signals was 0.89 for the neck area, 0.92 for the chest area, and 0.92 for the abdomen area.

**Figure 12:**
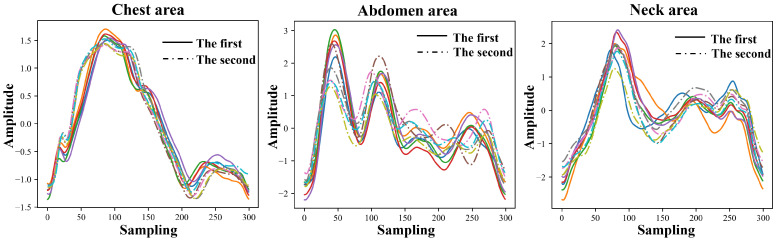
Pulse wave signals from different body parts of Subject 3 in two experiments conducted one month apart (randomly selected signals from 5 cycles in each experiment, identified using different colors).

Figure 12 illustrates the signal morphology of the same subject during two experiments conducted one month apart. It can be observed that although the similarity of the waveform morphology between the signals one month apart decreased, the key feature points of the pulse wave signals in various parts are not missing or changed, and the similarity of the overall contour and trend of the pulse wave signal is high enough.

Comparing the subjects in two experiments conducted one month apart, the overall morphological similarity of the pulse wave signals in various parts was as follows: the average similarity of the neck pulse wave signals was 0.84; the average similarity of the chest pulse wave signals was 0.87; and the average similarity of the abdominal pulse wave signals was 0.85.

**Figure 13:**
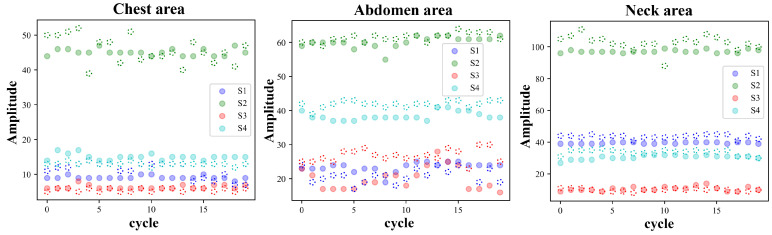
Pulse wave signals from the chest, abdomen, and neck area of Subject 1 in two experiments conducted one month apart (20 cycles’ characteristics randomly selected from each experiment, the solid circles represent the first experiment, while dashed lines represent the second experiment).

**Figure 14:**
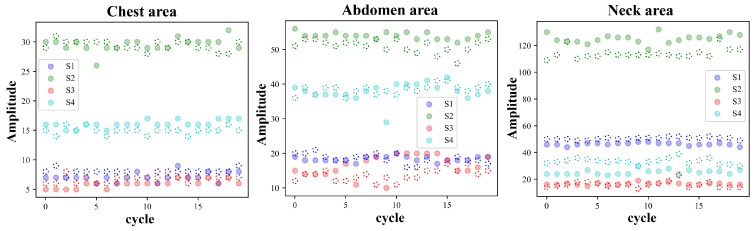
Pulse wave signals from the chest, abdomen, and neck area of Subject 2 in two experiments conducted one month apart (20 cycles’ characteristics randomly selected from each experiment, the solid circles represent the first experiment, while dashed lines represent the second experiment).

Taking Subject 1 and Subject 2 as examples, Figure 13 and Figure 14 show the changing trends of the four characteristics of each part of the two subjects during the two experiments. It can be observed that for a specific individual, the pulse wave characteristics of each part are basically stable during the measurement process in different time periods.

**Figure 15:**
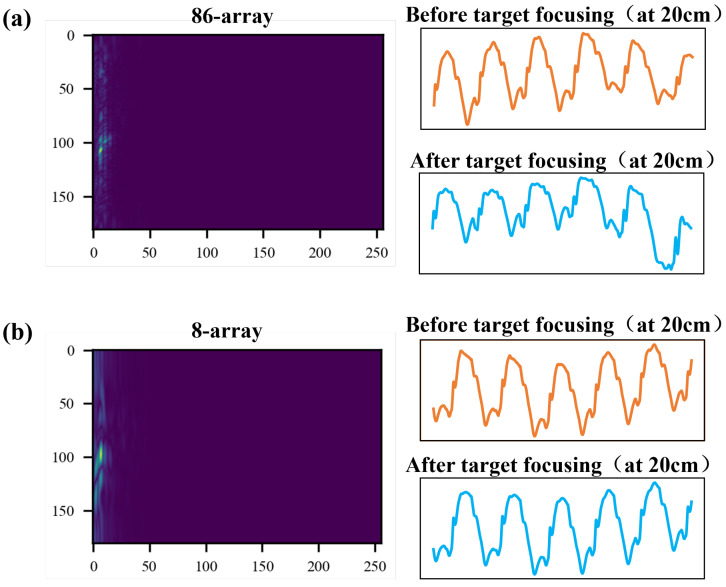
(**a**) Range–angle heat map of the 86-element antenna at a measurement distance of 20 cm and the chest signal before and after range–angle focusing; (**b**) range–angle heat map of the 8-element antenna at a measurement distance of 20 cm and the chest signal before and after range–angle focusing.

Next, we will discuss the impact of array size on signal acquisition, as well as the necessity and effectiveness of range–angle target positioning and focusing processing. Figure 15a shows the chest area signal obtained by the 86-array radar before and after range–angle target focusing and positioning at a distance of 20 cm from the chest; only the first 8 arrays are taken, and the chest area signal obtained before and after target positioning and focusing processing is shown in Figure 15b. It can be seen that when the measurement distance is 20 cm, the small array has little effect on the signal morphology, and the overall morphology and key features are relatively stable. In fact, at a measurement distance of 20 cm and 30 cm, the chest signal does not change much before and after target positioning and focusing. This is because in the case of short distance, the beam focusing area is small, and the signal in the non-target direction has less interference with the target direction signal; as the distance increases, the beam focusing area becomes larger, and the non-target direction falls within a narrower monitoring angle range, leading to greater interference with the target direction signal. Therefore, the farther the monitoring distance, the more necessary the range–angle positioning and focusing processing of the target is.

**Figure 16:**
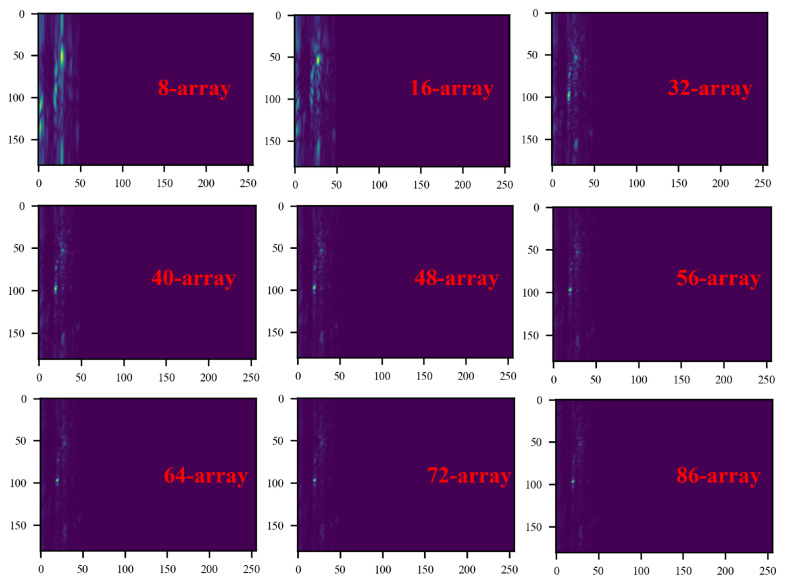
The corresponding range–angle heat map when the number of arrays increases from 8 to 86 (The greater the energy, the brighter it appears).

**Figure 17:**
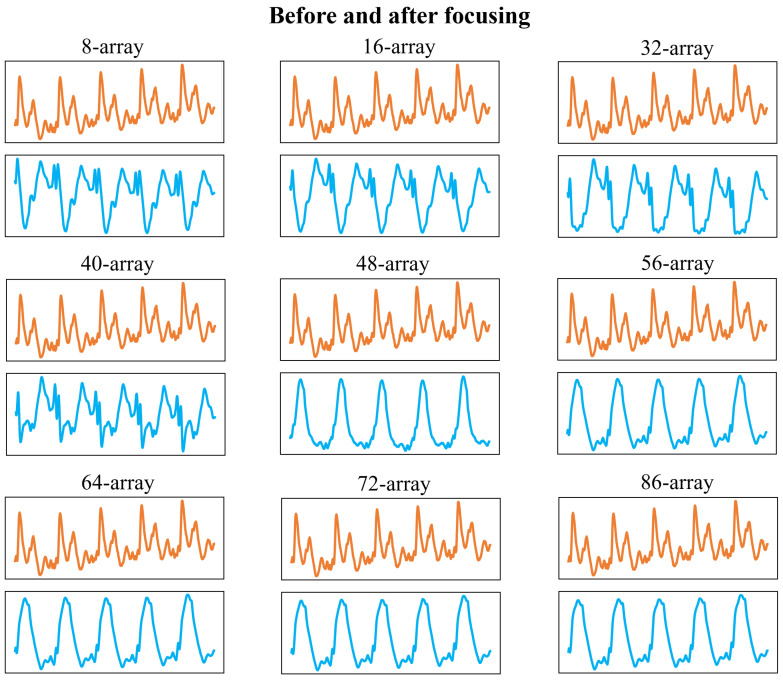
Chest signal morphology before and after target localization focusing when the number of arrays increases from 8 to 86 (Yellow and blue represent the signals before and after focusing, respectively).

Next, we will discuss the effect of array size on signal morphology at a longer monitoring distance. Figure 16 shows the range–angle heat map of the signal when the array size is gradually increased from 8 arrays to 86 arrays when the measurement distance is 70 cm. Figure 17 shows the comparison of the pulse wave signals before and after range–angle focusing when the radar is placed directly on the chest of the subject at a measurement distance of 70 cm. The larger the number of arrays, the clearer the positioning of the target in the corresponding range–angle heat map. When the number of arrays is small, even if the range–angle focusing algorithm is used, the correlation between the waveform morphology of the signal at 70 cm and that of the signal at 20 distances is less than 0.5. Starting from 48 arrays, the overall contour morphology of the signal significantly improves, but the key pulse wave features are not obvious. Starting from 56 arrays, the signal morphology has a high similarity with the signal at 20 cm, and the key features are more obvious. At this time, the beam width is approximately 25 mm. It can be seen that before the target range–angle positioning and focusing, the signal morphology of the chest has changed greatly compared to the signal at the close distance, and some pulse wave features have changed. However, after the target positioning and focusing processing, the signal morphology has a high similarity with the signal at the short distance (20 cm) measurement, and the key pulse wave features are retained.

It illustrates the superiority of a large array and the necessity of range–angle positioning and focusing processing when measuring at a longer distance. By performing these two processes simultaneously, the signal can maintain a more stable form.

**Figure 18:**
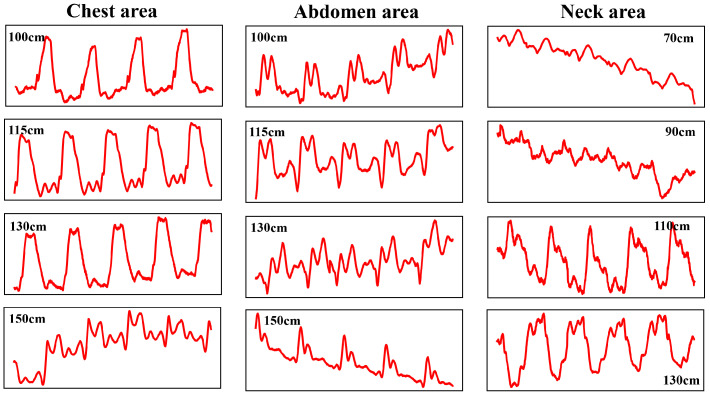
Signals of various body parts at different measurement distances using the 86 arrays and after focusing processing.

Next, the signals of various parts obtained after using the 86 arrays and the range–angle positioning and focusing algorithm at a longer measurement distance are further shown. Figure 18 shows the pulse wave signals of the chest, abdomen, and neck after being processed at different measurement distances.

As can be seen from Figure 18, at a measurement distance of 130 cm, the chest signal has a high correlation with the signal at a close distance, but the S1 feature is no longer obvious; the abdominal signal has a large change in overall shape at 150 cm; and in the neck area, at 90 cm, the overall shape of the signal and the pulse wave characteristics are no longer stable. This may be because the blood vessel monitoring area of the neck is smaller than that of the chest and abdomen, so it is difficult to accurately capture high-quality signals at a longer distance. Based on these test results, the limit value of the beam width can be preliminarily obtained, and then the relationship between the distance and the number of arrays can be inferred, providing a valuable reference for pulse wave monitoring based on array radar.

## 4. Discussion

Most of the previous work on non-contact pulse wave measurement based on radar mainly studies and explains the differences in the morphology of pulse wave signals, explaining the reasons for the inconsistency in the waveform morphology of pulse wave signals measured at different times. Without considering the influence of signal processing algorithms such as filtering and modal decomposition, the main reason for this phenomenon is that the radar monitoring area is larger, so it is difficult to accurately focus on the target area. Therefore, the echo signal contains mixed signals from multiple places, resulting in irregular signal morphology. As far as the author knows, no related research has been reported so far to quantitatively evaluate the repeatability and regularity of the morphology of pulse wave signals at different body parts measured by radar. We summarize two possible reasons in hardware and algorithms: in hardware, it comes from the limitations of the angular resolution of small-array radar; in algorithms, we also summarize an aspect that was easily overlooked by researchers before. Although some research works use beamforming algorithms, the use of this algorithm is based on the premise that the radar is not placed in alignment with the target. In most monitoring scenarios where the radar is facing the target, researchers mainly start from the energy of the range bin when selecting the target area, but ignore the consideration of angle. Directly extracting the target range bin signal means obtaining an omnidirectional signal of 0–180 degrees. The longer the measurement distance, the greater the impact on the signal shape. If only considering monitoring frequency indicators such as respiratory rate, heart rate, and pulse rate, the specific morphological details of the signal can be ignored so the signal can be obtained only according to the range bin; but if considering obtaining the pulse wave morphological characteristics for cardiovascular status or blood pressure and other indicators, more accurate positioning needs to be used to obtain a more reliable and stable pulse wave morphology.

Therefore, we used MIMO array radar and combined it with range–angle target focusing and positioning processing to obtain the arterial pulse wave signals of the subjects’ neck, chest, and abdominal areas, and analyzed the stability and repeatability of the signals in each body part during different measurement periods.

In addition, we further designed experiments to discuss the influence of array size and measurement distance on pulse wave acquisition. In this work, a 16 × 12 array radar is used. In actual application scenarios, such a large array may be not required. Therefore, we explored the array size required to obtain a pulse wave with stable morphology at different measurement distances, and gave the experimental results. Researchers can choose a suitable antenna array based on this result and their own measurement scenario requirements. This experimental work provides an effective reference solution for non-contact long-term measurement of arterial pulse waves based on mmWave radar.

This study also has several limitations: age, body shape, and health status may affect the accuracy of millimeter-wave radar measurements. The pulse wave signals may vary across different age groups; body shape could influence the radar’s ability to receive and demodulate pulse wave signals; individuals with certain health conditions, such as cardiovascular diseases, diabetes, and obesity, may exhibit different pulse waveforms and physiological characteristics. We plan to explore the performance of millimeter-wave radar alongside various health conditions in future studies to ensure its effectiveness in diverse populations. Furthermore, the widespread adoption of millimeter-wave radar technology still faces certain limitations, particularly in large-scale population studies and clinical trials. Key challenges include individual differences, data diversity, environmental adaptability, and ethical compliance. In order to address these challenges and promote the broad application of millimeter-wave radar technology in vital sign monitoring, we plan to conduct larger-scale population studies in future research, contributing to the further development and practical application of this technology.

## 5. Conclusions

The stability of pulse wave signals in various parts of the body is very meaningful for extracting stable pulse wave features to analyze cardiovascular conditions, blood pressure, and other indicators. The non-contact pulse wave measurement method brings the significant benefit of zero interference and can complete the monitoring without the user feeling, which makes its operation more suitable for non-medical users.

In this study, we used a 77 GHz mmWave MIMO array radar system to quantitatively evaluate the periodicity, repeatability, and waveform morphology similarity of non-contact pulse wave signals of different body parts. The measurement results show that the system, supplemented by the range–angle target positioning and focusing algorithm, can monitor periodic, repeatable, and stable pulse wave signals of different body parts. The neck, chest, and abdomen are proximal to the aorta, which are more suitable for characterizing cardiovascular conditions than peripheral arteries such as the wrist and fingertips. For six subjects, the pulse wave signals of different parts measured under different conditions achieved an overall morphological similarity of more than 0.84 and had stable pulse wave characteristics. In addition, we also verified that as the measurement distance increases, the using of a target positioning and focusing algorithm can obtain a more stable pulse wave morphology, which also illustrates the importance of this algorithm for pulse wave morphology analysis. Although the field of using radar to monitor vital signs has developed rapidly, most of the work is aimed at studying indicators such as heart rate and pulse rate. The existing systems and algorithms lack related work on the measurement of pulse wave signals. Therefore, we hope that our research can bring possible inspiration to this part of the work.

The experimental results of this study prove that the use of large-array millimeter-wave radar can monitor stable arterial pulse wave signals in a non-contact manner over a continuous, long period of time, and the experiment was designed to discuss the impact of the number of arrays and the measurement distance on the stable extraction of pulse waves, providing a reference value for pulse wave monitoring based on mmWave radar. This work is expected to provide a possible new solution for long-term monitoring of cardiovascular diseases in future research.

## Figures and Tables

**Table 1:** Antenna transmission parameter settings.

Parameter Settings	Value
Start frequency, fc (GHz)	77
Slope, S (MHz/µs)	100
Idle time (µs)	5
ADC start time (µs)	2
ADC sampling	256
Sample frequency (ksps)	7000
Ramp end time (µs)	35
Number of chirps per frame	12
Inter frame interval (ms)	5

**Table 2:** Similarity of pulse wave signals from different body parts of the subjects in the same experiment.

Subject	Correlation of Neck Signals	Correlation of Chest Signals	Correlation of Abdomen Signals
Subject 1	0.94	0.93	0.95
Subject 2	0.85	0.92	0.90
Subject 1	0.88	0.90	0.89
Subject 1	0.87	0.92	0.90
Subject 1	0.90	0.94	0.97
Subject 1	0.91	0.90	0.92

## Data Availability

The data that support the findings of this study are available upon reasonable request from the corresponding author. The data are not publicly available due to privacy or ethical restrictions.
